# The Anatomy of Phi Thickening Development in *Brassica oleracea* Roots

**DOI:** 10.3390/plants15203162

**Published:** 2026-10-15

**Authors:** David A. Collings, Maketalena Aleamotuʻa, David W. McCurdy

**Affiliations:** 1 School of Biological Sciences, The University of Canterbury, Christchurch 8140, New Zealand; 2 School of Environmental and Life Sciences, The University of Newcastle, Callaghan, NSW 2308, Australia; maketalena.akauola@gmail.com (M.A.); david.mccurdy@newcastle.edu.au (D.W.M.); 3 Research School of Biology, The Australian National University, Canberra, ACT 2601, Australia; 4 School of Agriculture, Tonga National University, Nukuʻalofa, Tonga

**Keywords:** *Brassica oleracea*, Casparian strip, lateral roots, phi thickenings, root development, secondary cell walls

## Abstract

In response to jasmonic acid, inner cortical cells in *Brassica oleracea* roots sequentially form two different secondary cell wall arrays. Initially, these cells develop phi thickenings, thick bands looped around the radial walls that are coordinated between adjacent cells to form a reinforcing scaffold. These thickenings develop later than the formation of the Casparian strip and protoxylem. Subsequently, inner cortical cells develop a reticulate network of delicate thickenings on their inner face adjacent to the endodermis. While phi thickenings initially develop in a single layer in the inner cortex, we demonstrate that multiple rings can develop in two ways, either through the occasional development of isolated thickenings in individual cells in the middle cortex, or through cell divisions in the endodermis that generate a new inner cortical layer which forms extensive phi thickenings. We also show that the phi thickening network in developing lateral roots remains separate from the network in the primary root, even as the Casparian strip re-forms a contiguous network. These anatomical observations have implications not only for understanding the developmental pathways leading to phi thickening formation but also for investigating the rare example of two different secondary wall structures being deposited in the same cell type.

## 1. Introduction

Phi thickenings are secondary cell wall bands that develop in the radial walls of cells in the root cortex, a location that would normally only exhibit a thin, primary cell wall. The positioning of these wall thickenings is coordinated between adjacent cells and, when combined, forms a scaffold around the vascular tissue. Phi thickenings have been reported in a diverse range of angiosperm and gymnosperm families, including several agriculturally and horticulturally important species [1,2,3,4,5]. While phi thickenings were first observed more than 100 years ago [6,7], it remains unclear as to whether the structures represent an example of conservation or convergent evolution [5]. It is also unclear what functions phi thickenings might perform in roots. While roles in the regulation of nutrient uptake, in a manner analogous to the endodermis, and in controlling interactions with mycorrhizae and root pathogens have been suggested, a mechanical role in strengthening or stiffening the root seems more likely [3,4].

We broadly define phi thickenings as lignified and thickened secondary walls that form in localized bands around root cortical cells [3]. This definition includes classical phi thickenings in which the wall thickenings form a simple rectangle in the radial walls, a pattern conserved in many angiosperms and gymnosperms, but also includes more randomized banding patterns seen in various species, including some orchids and gymnosperms [5]. The Brassicaceae also show complex phi thickenings in which thick, rectangular phi thickening bands in the radial walls of inner cortical cells are supplemented by a network of thinner secondary walls that spreads across the inner face of these cells where they face the endodermis. First reported and illustrated by Woronin [8] (Figure S1), we refer to these secondary walls as the reticulate network because of the polygonal arrays that develop in *Brassica* [5,9,10]. However, the specific patterns formed by this network vary between species within the family [9,11], ranging from the polygonal array present in *Brassica* [5,9,10] to the almost completely thickened and lignified inner wall in the heavy metal hyperaccumulator *Noccaea caerulescens*, structures which have been referred to as peri-endodermal thickenings [12,13,14]. The ability to form a reticulate network seems to be broadly ubiquitous within the Brassicaceae, with *Arabidopsis* species being an exception to this, with no reticulate network having been observed [5]. As with phi thickenings, the function(s) of the reticulate network remain unknown.

Multiple stimuli can trigger the development of both classical phi thickenings and the reticulate network in Brassicaceae roots. Salt treatments result in phi thickening and reticulate network formation in both radish [15] and *Brassica oleracea* roots [16,17], but this development is not dependent on salt, per se, but rather results from a more general osmotic effect in which both abscisic acid (ABA) and gibberellic acid (GA) act upstream from jasmonic acid (JA) to control thickening development [10]. JA also triggers phi thickening development throughout most species within the Brassicaceae as well as in the Fabaceae (*Senna occidentalis*) and Geraniaceae (*Geranium robertianum* and *Pelargonium australe*) [5]. In addition to the osmotic induction pathway, mechanical effects can also trigger the formation of phi thickenings with increased hardness and roughness of the surface across or through which the roots grow, also triggering phi thickening formation, again acting through JA [10].

In this study, we sought to answer several specific developmental questions concerning phi thickening development, including better identifying where these structures form in comparison to the development of other lignified tissues such as the xylem and Casparian strip. Furthermore, from the earliest descriptions of phi thickenings in *Brassica*, it has been apparent that phi thickenings can develop in more than one layer and occupy both the inner cortex adjacent to the endodermis as well as the next cortical layer, with both cell layers developing a reticulate network. In their investigations of the ability of 80 mM sodium chloride solutions to induce phi thickenings in *Brassica oleracea* roots grown in hydroponics, Fernández-García and colleagues [17] observed that roughly a third of the roots formed a second ring of phi thickenings, with the reticulate network being present on the inner face of both rings. Woronin also reported multiple layers of phi thickenings with the reticulate network generally limited to the inner face of these cells [8] (Figure S1). The development of these multiple layers has not, however, been investigated. How phi thickenings develop at the junction between primary and lateral roots is also unclear, but because lateral roots form from divisions initiated in the pericycle, inside the endodermis, lateral roots must emerge through the phi thickening layer. In *Malus* (apple) roots, the phi thickenings of the lateral root do not connect to the phi thickenings in the primary root and seem to form a structurally separated network [18], which contrasts with the Casparian strip, which re-forms following lateral root formation. Understanding the development of multiple phi thickening layers and phi thickenings in lateral roots may provide insight into the cellular pathways that drive phi thickening production within roots, and the functions that thickenings perform.

## 2. Results

### 2.1. The Spatial Distribution of Phi Thickening Development and Endodermal Suberization

**Figure 1:**
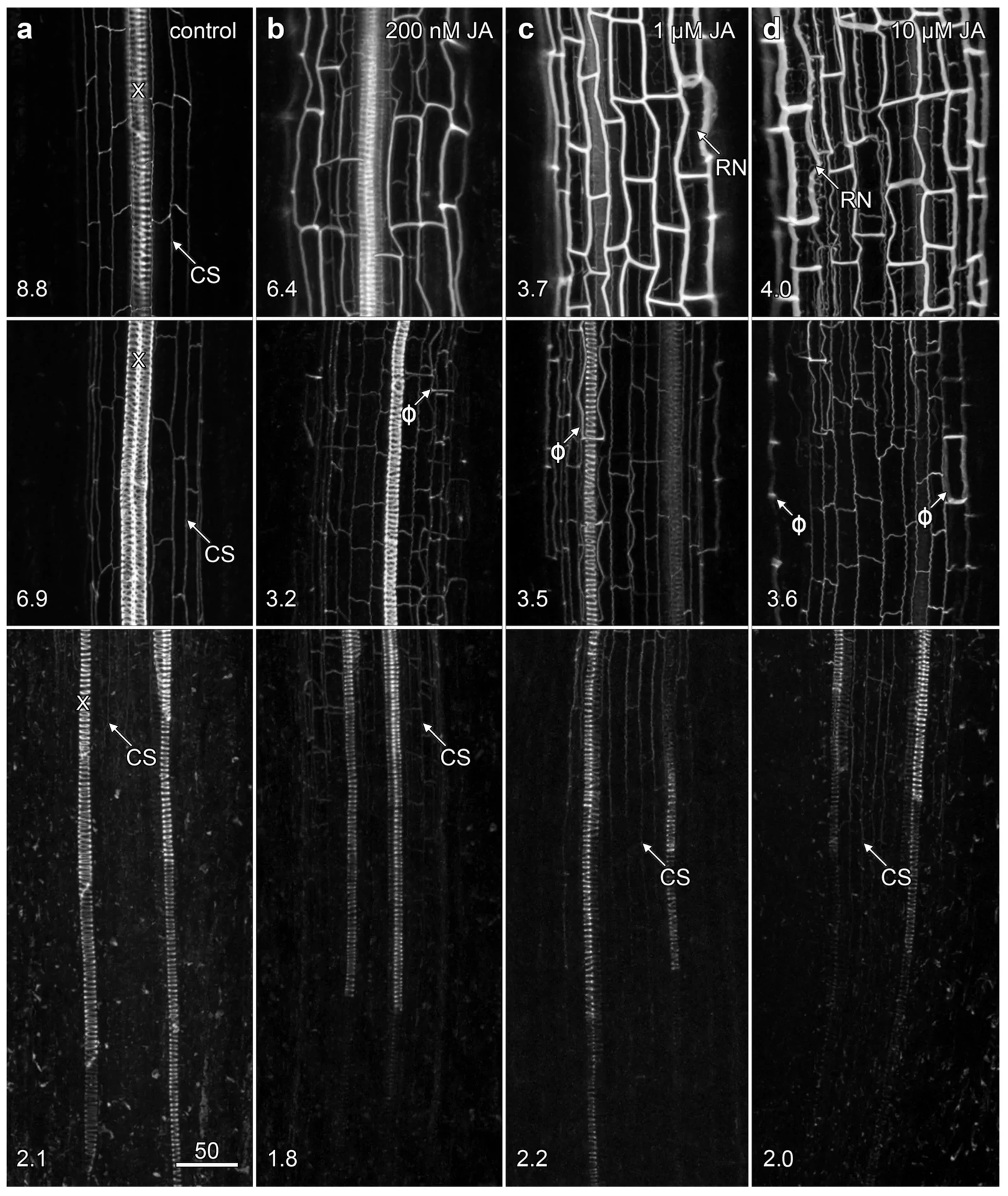
Phi thickening development in *Brassica* root tips was screened by confocal imaging of basic fuchsin-stained lignin, with maximum projection images presented at three separate distances from the root tip. The bottom row shows the initial development of the xylem and Casparian strip (CS), while the central row shows the first development of phi thickenings (ϕ), with this development initially being patchy. Xylem vessels (X) are shown in (**a**). The top row shows the first visible reticulate network (RN) among the nearly complete phi thickening network. Numbers on images indicate distances from the root tip in millimeters. (**a**) Control roots. (**b**) 200 nM JA. (**c**) 1 µM JA. (**d**) 10 µM JA. Scale bar in (**a**) = 50 µm for all images.

**Figure 2:**
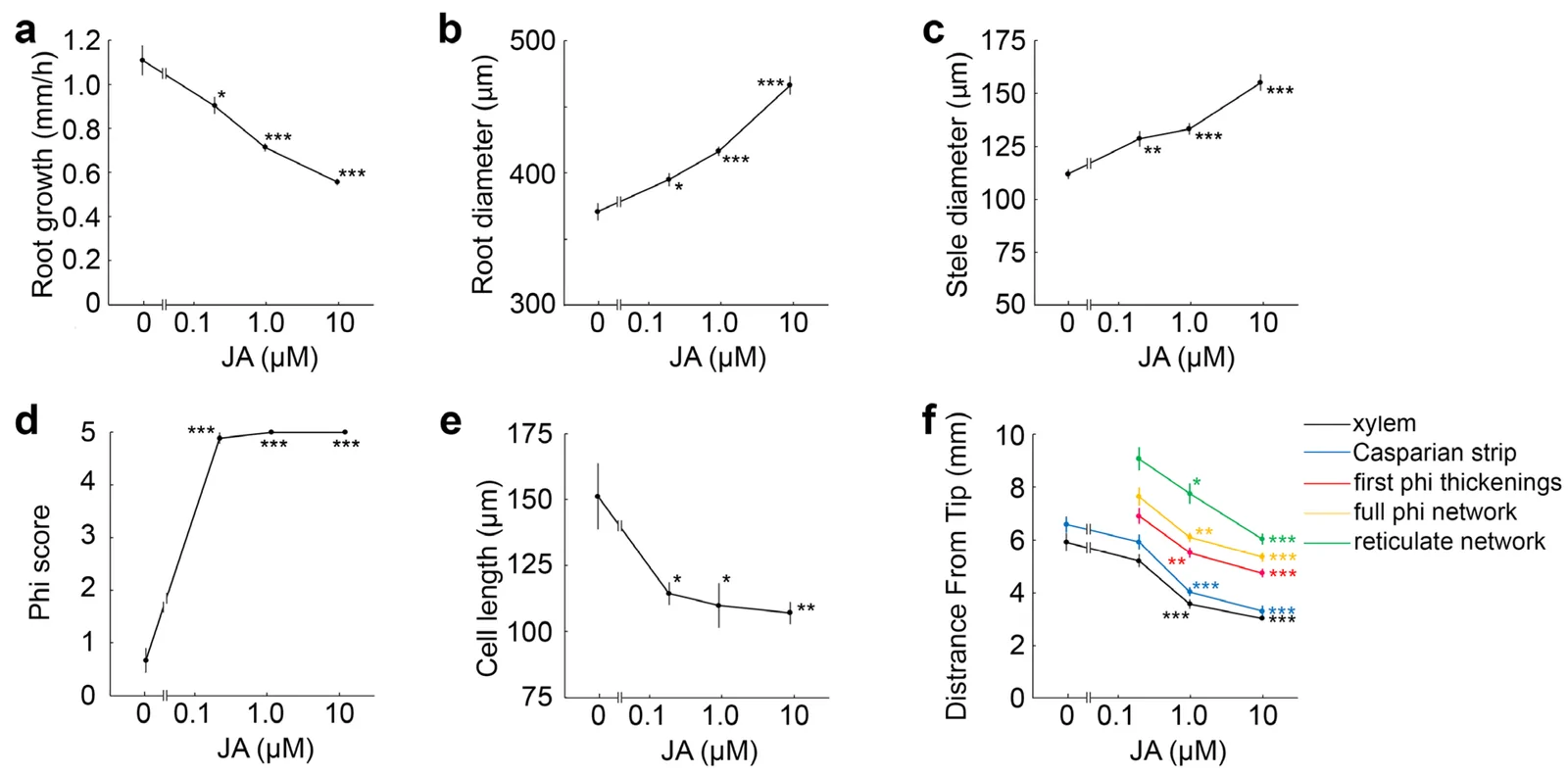
Quantification of JA effects on root development, including the induction of phi thickenings. (**a**) Root elongation rates. (**b**) Root diameter. (**c**) Stele diameter. (**d**) Phi thickening scores. (**e**) Cell lengths, measured in inner cortical cells. (**f**) Distance from the root tip where different structural components were first detected through lignin labeling with basic fuchsin: xylem, Casparian strip, phi thickenings and the reticulate network. The graph also indicates where the phi thickenings first reached their maximum density. Data are means ± standard errors for n ≥ 9 roots. Values significantly different from the control (*t*-test) are shown, except for the development of phi thickenings and reticulate network, which were absent from control roots: * *p* < 0.05; ** *p* < 0.005; *** *p* < 0.0005.

In our previous study of phi thickening development in *Brassica oleracea*, we suggested that development of the Casparian strip and phi thickenings occurred concurrently at approximately the same distance from the root tip [10]. We have now refined this developmental sequence (Figure 1 and Figure 2). We investigated root development in response to growth on plates containing JA concentrations between 200 nM and 10 µM. Confocal imaging showed that the Casparian strip and first protoxylem vessels develop roughly concurrently in both control (Figure 1a) and JA treatments, while the first phi thickenings induced by JA develop further from the root tip (Figure 1b–d). The development of phi thickenings was initially patchy, with gaps apparent in their development even at 10 µM JA (Figure 1d). These gaps were eventually filled in, although occasional phi thickening-free cells remained present even as far as 20 mm from the root tip (Figure S2).

We quantified the development of roots in response to growth on JA from montages of widefield fluorescence and DIC images collected over the first ~20 mm of roots. Compared to controls where roots grew at about 1.1 mm·h^−1^, JA significantly reduced the rate of root elongation by 19, 36 and 50% respectively (Figure 2a), and significantly increased stele and root diameters (Figure 2b,c). Consistent with reductions in root elongation, inner cortical cell lengths, measured in cells containing phi thickenings, were also significantly reduced in JA treatments (Figure 2e). Lignin fluorescence from xylem was first detected about 5 mm from the root tip, apical to the detection of the first lignin fluorescence from the Casparian strip. As JA concentrations increased, both xylem and the Casparian strip were detected significantly closer to the root apex, but even at 10 µM JA, these developed about 3 mm from the root apex (Figure 2f).

In these rapidly growing roots, growth on JA resulted in strong induction of phi thickenings even at JA concentrations as low as 200 nM (Figure 2d). At this concentration, phi thickenings were first visible 6.9 ± 0.3 mm from the root tip (range 5 to 8 mm) when grown on 200 nM JA, significantly further from the tip than the first visible xylem (5.2 ± 0.2 mm) and Casparian strip (5.9 ± 0.3 mm) (*p* < 0.005 for both comparisons), and the network was generally complete by 6 to 9 mm. The reticulate network was first detected between 6 and 10 mm from the root tip in the JA treatments (Figure 2f). Increasing JA concentration resulted in the first formation of phi thickenings and the reticulate network occurring closer to the root tip. These observations are consistent with the slowing of root elongation caused by JA. With root growth of between 1.1 mm·h^−1^ in the control, decreasing to ~0.6 mm·h^−1^ in the highest tested JA concentration, the difference between the location of initial observation of the xylem and Casparian strip of about 0.3 to 0.6 mm corresponds to less than an hour, while the difference in location between the formation of the phi thickenings and reticulate network (about 1.5 mm in the lower JA concentrations) corresponds to several hours of growth. This distance would be consistent with the time delays previously reported for the formation of the reticulate network [5].

### 2.2. The Formation of Double Rings of Phi Thickenings

**Figure 3:**
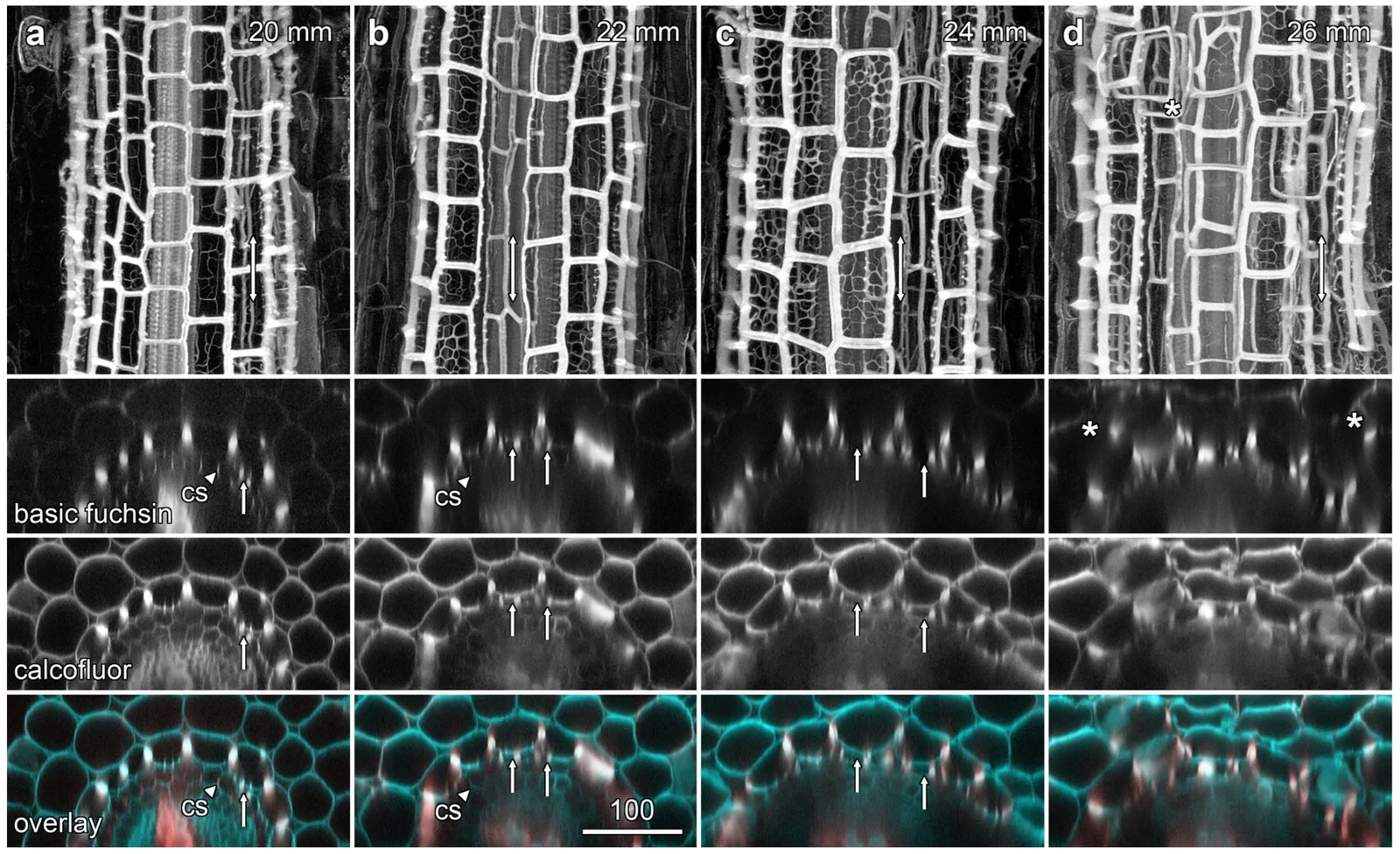
The development of multiple rings of phi thickenings. Cross sections (lower 3 rows of images) generated in ImageJ using the reslice function show basic fuchsin labeling of lignin, calcofluor white labeling of cell walls and an overlay of calcofluor (cyan) and basic fuchsin (red). The upper panels show longitudinal sections and basic fuchsin-stained lignified walls. In cross sections, arrows show the extra internal layer of phi thickenings while asterisks represent extra external phi thickenings. In longitudinal sections, the double-headed arrows indicate extra files of thickenings internal to the complete phi thickening ring, whereas the asterisks mark single cells with thickenings that are external to the general phi thickening layer. The Casparian strip (cs) is indicated with arrowheads in (**a**,**b**) but was not clear in older parts of the root. Scale bar in (**d**) = 100 µm for all images. See also: Video S1.

**Figure 4:**
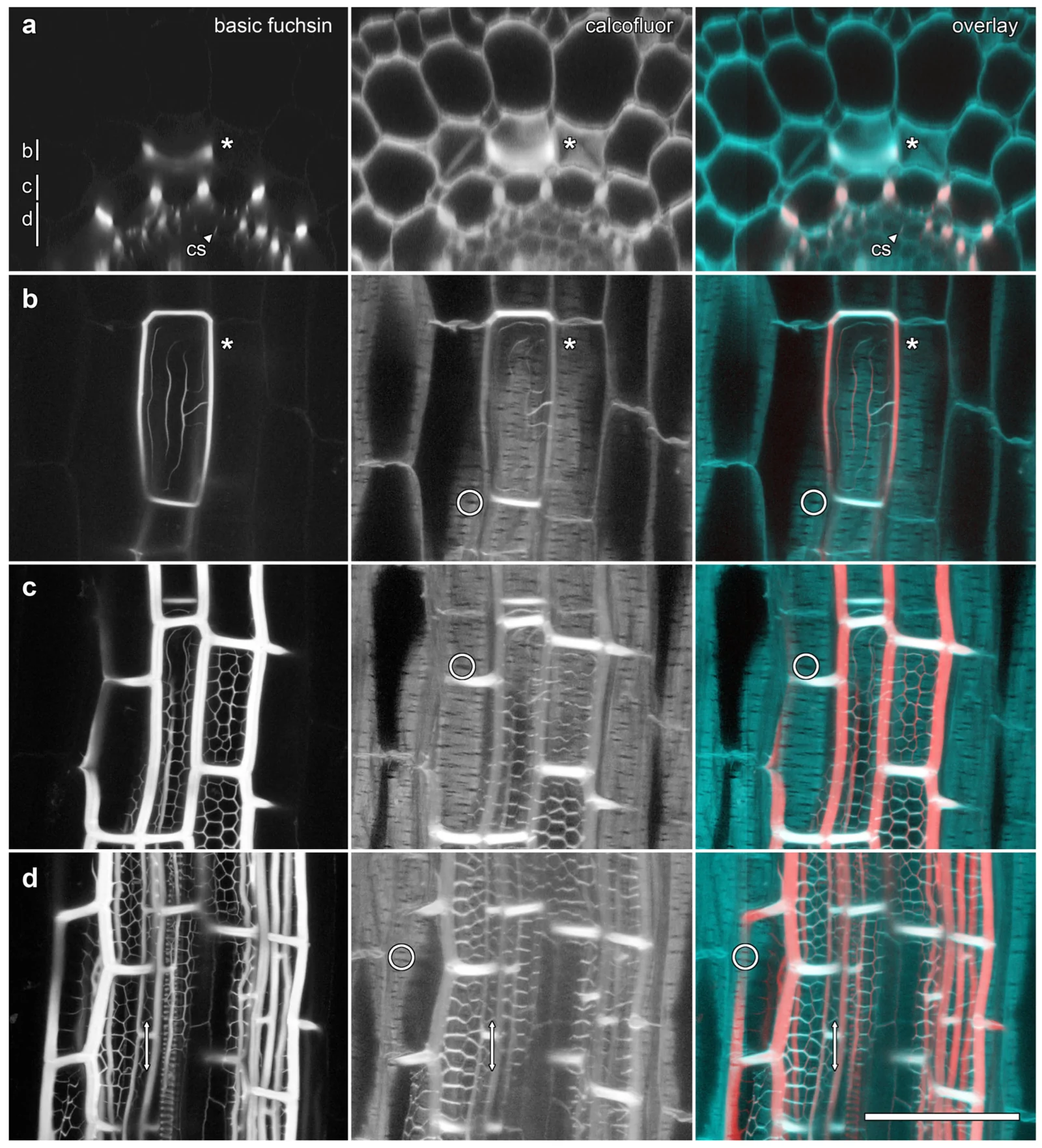
The development of multiple rings of phi thickenings. (**a**) Cross sections generated in ImageJ using the reslice function (upper row) show basic fuchsin labeling of lignin, calcofluor white labeling of cell walls and an overlay of calcofluor (cyan) and basic fuchsin (red). (**b**–**d**) Maximum projections of longitudinal sections taken at three different depths into the root, at locations indicated in (**a**) with letters and bars. The asterisk (**b**) marks a single extra cell with phi thickenings, while the double-headed arrow (**d**) shows extra files of phi thickening-containing cells internal to the main ring of phi thickenings. The Casparian strip (cs) is indicated with arrowheads. The dark, horizontal bands present in the calcofluor images (circled) are pit fields. Scale bar in (**d**) = 100 µm for all images. See also: Video S2.

Because understanding how multiple rings of phi thickenings originate may be informative on the mechanism through which the thickenings are formed, we assessed confocal image stacks, generating 3D image reconstructions and re-slicing data to calculate both cross sections and radial longitudinal sections. We conclude that there are two different ways in which multiple thickening rings can be formed, with these being more common in roots grown on 10 µM JA than either 200 nM or 1 µM JA, where extra layers were extremely rare (Figure 3 and Figure 4). While JA-induced phi thickenings begin to form between 3 and 5 mm from the root tip (Figure 1 and Figure 2), imaging along *Brassica* roots demonstrated that the formation of extra layers of phi thickenings commences between 10 and 20 mm from the tip. First, individual cells in the middle cortex, immediately outside the inner cortex, occasionally developed phi thickenings, but if they do, then they are usually isolated and not part of a network (Figure 3d, Video S1, asterisks). Second, discrete files of extra phi thickenings (Figure 3a–d, Video S1, double-headed arrows) arise through divisions of the endodermal cells, as demonstrated in radial longitudinal reconstructions of the root. The result of endodermal divisions is a second layer of smaller cells inside of the initial inner cortical layer that also develop phi thickenings. The distribution of cells in this new cell layer matches the endodermal cells when viewed in cross sections (Figure 3a–d, arrows).

This ability for high concentrations of JA to trigger multiple layers of phi thickenings is also shown in Figure 4 and Video S2, in which the orthogonal reconstructions are accompanied by maximum projections at three different depths within the root that highlight isolated mid-cortical cell phi thickenings (Figure 4b), the regular phi thickening network in the inner cortex (Figure 4c) and the formation of a new layer of phi thickenings through the division of the endodermis (Figure 4d).

### 2.3. Phi Thickening Development and Lateral Roots

The formation of lateral roots begins with cell divisions that are initiated within the pericycle, inside both the endodermis with its Casparian strip and the inner cortex with phi thickenings [19,20]. Lateral root emergence through the root cortex must, therefore, disrupt both the Casparian strip in the endodermis and the phi thickenings in the inner cortex.

**Figure 5:**
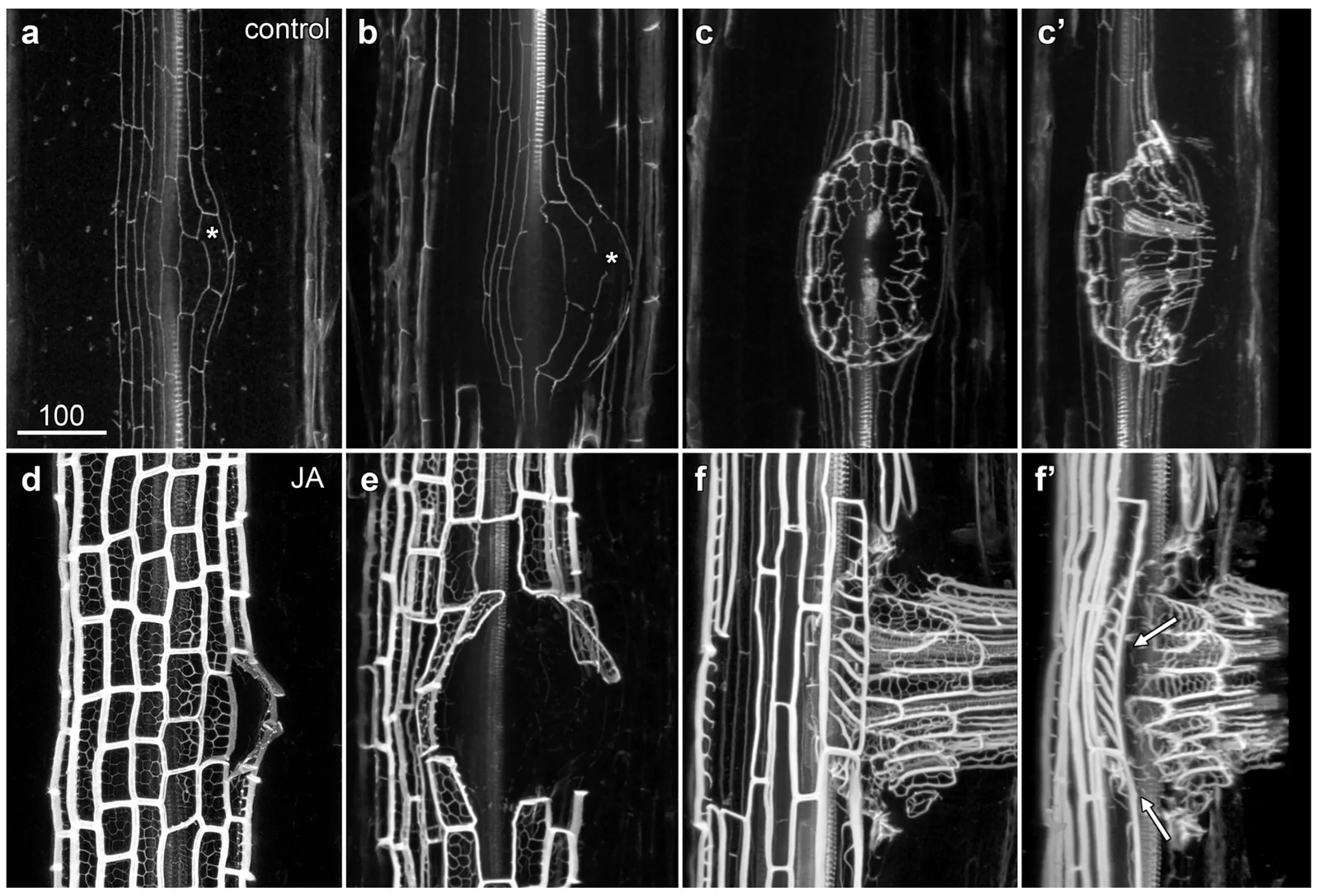
Casparian strip and phi thickening organization during lateral root development. (**a**–**c**) Uninduced root. (**d**–**f**) Root grown on JA (1 µM). (**a**,**d**) Lateral root initial only just formed. Fractures in the lignified Casparian strip had already developed (asterisks). (**b**,**e**) Lateral root extension to the surface of the primary root. (**c**,**f**) Older lateral root. Note that the phi thickenings in the lateral root form a separate network from the phi thickenings in the primary root, separated by a gap (arrow) in which the only lignified tissue is the Casparian strip ring. See also: Video S3. Images are maximum projections of confocal optical stacks of basic fuchsin-stained lignin, with the stacks to generate images (**c’**,**f’**) rotated through 45° to provide an oblique view. Scale bar in (**a**) = 100 µm for all images.

Our observations of lateral root development in *Brassica* roots demonstrate that instead of the loss of secondary cell walls during lateral root emergence, the phi thickening layer is instead mechanically disrupted by emerging laterals (Figure 5). We first investigated the continuity of the Casparian strip in uninduced *Brassica* roots. The Casparian strip, visible through lignin staining, initially curved over the developing lateral root primordia but even at this early stage, gaps had developed either through being stretched and broken (or, possibly, through localized degradation) (Figure 5a, asterisk). As the emerging lateral root enlarges, the Casparian strip ruptured, with further breaks in continuity developing (Figure 5b, asterisk). However, once the lateral root has emerged from the primary root, a new endodermis develops originating from cells in the pericycle, and these cells develop a new Casparian strip that connects to the original Casparian strip, with cells at the base of the lateral root forming a net-like array of Casparian strips when viewed end-on (Figure 5c; Video S3). These observations agree with the developmental sequence previously described in *Arabidopsis* [19].

When the development of laterals in roots containing phi thickenings was assessed, a different sequence was observed. While phi thickenings initially stretched somewhat over developing primordia (Figure 5d), the thickenings soon ruptured, dramatically separating along the primary cell wall and middle lamella (Figure 5e). In mature lateral roots, the phi thickenings did not connect to the thickenings present in the primary root (Figure 5f,f’), although this was best observed in 3D rotations (Video S3).

## 3. Discussion

In our study, we have further characterized the structural organization of *Brassica oleracea* phi thickenings following induction with JA. Understanding phi thickening organization has implications for identifying both their functions and the developmental pathways that lead to their formation. We have shown that phi thickenings develop close to the root tip, but that their development is several millimeters further away from the tip than the initial lignification of the protoxylem and Casparian strip, and thus several hours later. The relationship between the formation of thickenings, protoxylem and the Casparian strip is similar at different JA concentrations, with the formation of all these lignified structures moving towards the tip at higher JA concentrations, and as root growth slows. Additionally, we have demonstrated how phi thickenings can develop in multiple layers and confirmed that thickening networks in lateral roots fail to connect with the thickening network in the primary root. These observations have implications for understanding the pathways through which phi thickenings develop in *Brassica*, in other species in the Brassicaceae, and in angiosperm and gymnosperm species that form phi thickenings.

### 3.1. The Formation of Multiple Rings of Phi Thickenings May Provide Clues on Phi Thickening Signaling Pathways

We have used confocal microscopy and 3D image reconstructions to confirm that *Brassica oleracea* roots can develop multiple rings of phi thickenings. Our imaging demonstrated that these layers can arise through two distinct pathways, these being through the development of thickenings in the middle cortex, often unconnected to thickenings in adjacent cells, and through the division of the endodermis, with the outer cells arising from this division then forming a connected network of thickenings. While the isolated extra phi thickening containing cells formed outside the inner cortex are typically larger than the inner cortical cells, often in length and nearly always in radial dimensions, the connected extra cells formed from divisions of the endodermis are typically narrower and thinner in the radial orientation than the inner cortical cells. Divisions within the endodermis to form an extra cortical layer have previously been reported in Arabidopsis, with these divisions beginning after about a week of root growth and controlled by gibberellic acid [21,22]. We have not observed these multiple layers of phi thickenings in other species within the Brassicaceae, although roots in other Brassicaceae species have not been observed in as much detail as *B. oleracea*. However, multiple rings were observed in cross sections through *B. oleracea* roots in which phi thickenings were induced with saline hydroponics treatments [16,17], and the initial description of phi thickenings in *Brassica* roots described occasions where thickenings were present in two layers outside the endodermis [8]. Nevertheless, only by using 3D analyses can we fully appreciate the development of the multiple rings of phi thickenings and assess their origins.

Phi thickenings in the root cortex are traditionally split into three classes based on the location in which the thickenings first develop, with Type 1 thickenings present in the inner cortex, Type 2 being subepidermal and Type 3 thickenings present in the central layers of the cortex [1,2,3,5]. By this definition, *Brassica* roots begin by forming Type 1 thickenings but, by forming multiple layers of thickenings, end with a combination of Type 1 and Type 3 thickenings. *Ginkgo* initially forms normal Type 1 phi thickenings but then develops some mid-cortical thickenings [23], with these generally being isolated as seen in *Brassica* [5]. Similarly, thickenings begin as sub-epidermal Type 2 in *Geranium* and *Pelargonium* [24,25,26], but there are suggestions that they can develop further into the middle cortex in older regions of the root [24]. Instead of blurring the descriptions of the thickenings by saying that the *Brassica* (and *Ginkgo*) have both Type 1 and 3 thickenings, and Geranium has Type 2 and 3 thickenings, we believe that the definition of Type 1 through Type 3 thickenings might be redefined based on where phi thickening development is initiated.

### 3.2. Phi Thickening Networks in Lateral Roots Do Not Connect to Thickenings in Primary Roots

Lateral root development commences when cell divisions are initiated in the pericycle [19,20], with subsequent lateral root development resulting in mechanical disruption of the Casparian strip that links adjacent cells within the endodermis [27]. Subsequently, the Casparian strip re-forms around the lateral root, with cells developing a new strip that connects the Casparian strip in the lateral root and the Casparian strip in the primary root [19,28,29]. This development regenerates a continuous barrier to solute movement between the central stele and the root cortex. However, the formation of lateral roots also leads to the mechanical separation of the inner cortical cells, separating along the middle lamella (Figure 5), and the phi thickening network in the lateral root fails to connect with the phi thickening network in the primary root. In their study of phi thickenings in apple roots, Weerdenburg & Peterson [18] reported that “Lateral root primordia emerged through the phi layer by means of openings in the layer resulting from the removal of entire phi thickenings from the cortical cells” and concluded that “enzymatic digestion of the phi thickening rather than its mechanical disruption evidently occurred.” While the observations of phi thickenings being discontinuous between lateral and primary roots of *Brassica* matched the earlier reports in apple roots [18], the fracturing of the thickening network is different to the previously reported ‘loss’ of the lignified cell walls. This developmental sequence does, however, match the more recent study by Kováč et al. [14] in roots of *Noccaea caerulescens* where peri-endodermal thickening networks of the primary and lateral roots remain separate.

### 3.3. Phi Thickening Functionality

There have been no demonstrations of the function(s) of either the phi thickenings or the reticulate network. Thickenings have been suggested to regulate nutrient flows, aiding the function of the Casparian strip, control biotic interactions with mycorrhizae and pathogens, and to mechanically strengthen roots [2,3,4]. We have suggested previously that a mechanical role in strengthening or stiffening the root seems more likely [3,4]. Similarly, the reticulate network has been suggested to act as a form of transfer cell [2,13], although we have questioned this concept for several reasons, including that the amount of extra membrane provided would be limited, and because the lignification of the reticulate network walls is most unusual for transfer cells, as it would limit nutrient diffusion [4]. Three aspects of the results presented in this study suggest that the role of the thickenings is unlikely to be in controlling nutrient flows within the root. First, phi thickenings develop several millimeters further from the root tip, and thus several hours after the initial lignification of the Casparian strip, showing that a diffusion barrier is already in place when the thickenings form. Second, during the formation of lateral roots, which develop from the pericycle and break through both the endodermis and phi thickenings of the inner cortex, the Casparian strip reconnects to form a continuous diffusion barrier, whereas the phi thickenings fail to do so, forming a separate network. And third, the presence of occasional gaps in the phi thickening network also suggests that thickenings do not primarily function in the control of solutes and water movement. In our investigations of phi thickenings, we have not yet compared the development of phi thickenings to the subsequent suberization of the endodermis, a process that further strengthens the diffusion barrier because our standard clearing protocol, which uses potassium hydroxide and heat extraction, results in the alkaline hydrolysis of suberin [30].

With the hypothesis that phi thickenings act as a diffusion barrier seemingly unlikely, and with the hypothesis that phi thickenings regulate biotic interactions also being unlikely (thickenings can be induced in sterile conditions, and the Brassicaceae typically do not have mycorrhizal interactions [31]), we are left with the idea that the thickenings strengthen the root. This concept, first proposed more than 150 years ago [32], has not been tested, but the ability to control phi thickening development with JA makes such mechanical tests possible, and experiments testing this hypothesis have commenced.

### 3.4. Phi Thickening Signaling Pathways

**Figure 6:**
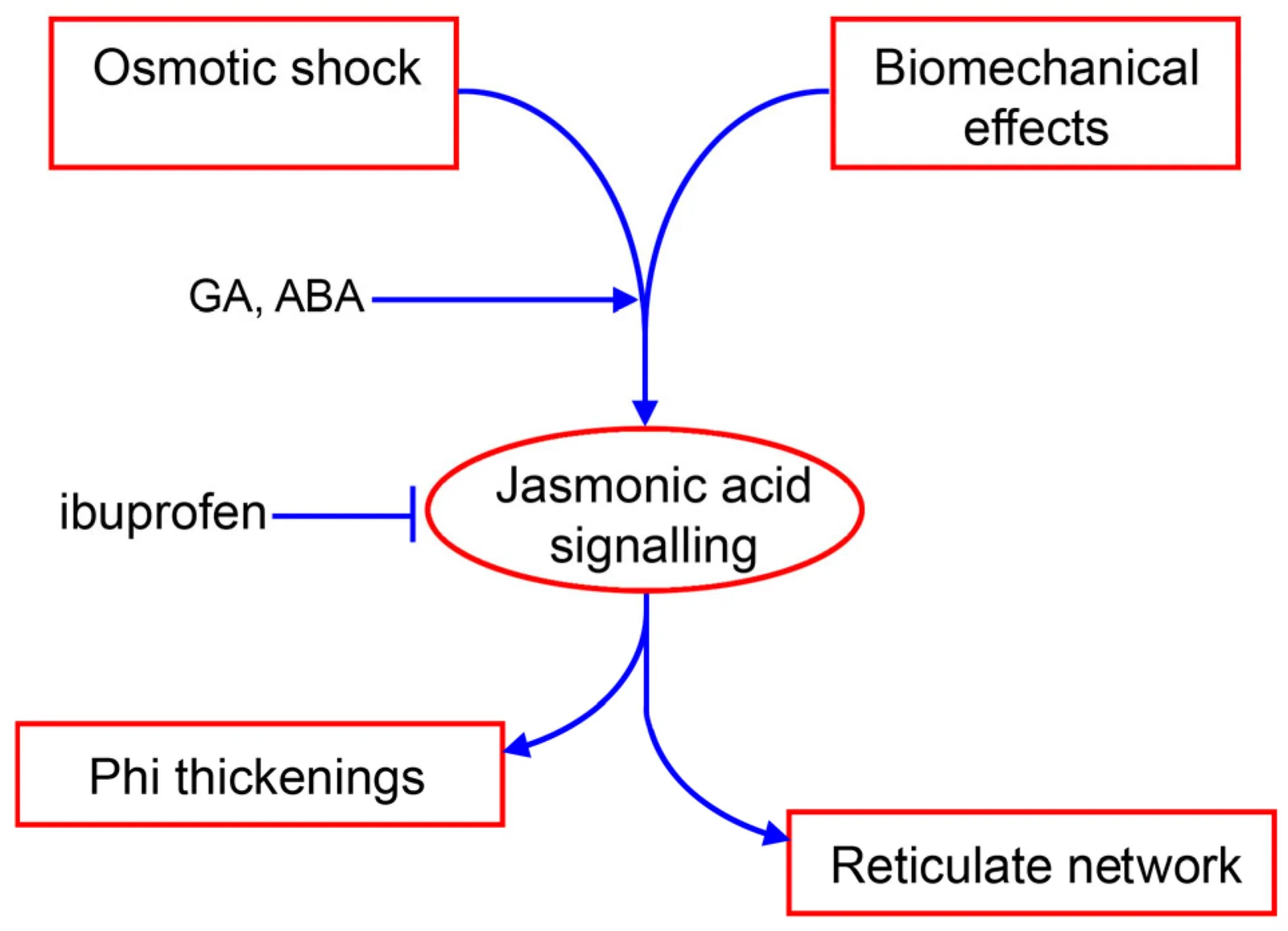
An updated model showing JA’s role in the induction of phi thickenings and the reticulate network, modified from [10]. ABA = abscisic acid, GA = gibberellic acid.

We previously proposed a model for the signaling pathway leading to phi thickening formation [3]. We now modify this model in two ways. First, we place osmotic shock and biomechanical effects on the same branch upstream from JA induction: we previously demonstrated that osmotic shock on its own was insufficient to trigger strong phi thickening induction, and that this only occurs in rapidly growing roots following salt treatments [10]. Indeed, this observation suggests that the signaling pathways involved in the biomechanical effects, which were not previously studied, should be investigated further. Second, we now show phi thickenings and the reticulate network as separate outcomes of JA signaling because of the differences of several millimeters in where these structures form compared to the root tip (Figure 6). This distance corresponds to a delay of several hours in the formation of the reticulate network compared to the thickenings, which matches previous observations of the timings of the formation of the two networks [5].

We previously proposed that signaling from the endodermis in the form of small diffusible peptides might explain phi thickening induction [3]. This type of signaling is important in the development of the Casparian strip [33,34], but also in the formation and maintenance of the root apical meristems [35], nodulation in legumes [36,37] and root hair initiation and development [38,39]. In all these cases, the peptides are detected by leucine-rich repeat receptor kinases. *Arabidopsis thaliana*, which does not induce phi thickenings [5], contains ~225 leucine-rich repeat receptor kinases (LRR-RKs) [40], most of whose functions remain unknown, while *Brassica oleracea* contains more than 300 LRR-RKs [41] and the tetraploid *B. napus* contains about 440 [42]. Our observation that phi thickenings can form multiple rings would be consistent with a model based on a diffusible peptide originating from the endodermis being sensed by an LRR-RK, with this strongly inducing thickenings in any new inner cortical cells formed from divisions of the endodermis, but only sporadically inducing in the middle cortex because of the distance that the signal needs to diffuse.

### 3.5. Conclusions and Future Directions

Our detailed anatomical characterizations will form an important basis for multiple ongoing studies concerning the signaling pathways leading to the sequential induction of two different secondary cell walls, the phi thickenings and the reticulate network. This ability for a single cell type to form two distinct secondary walls is unusual. While developing secondary walls in tracheids show periodic changes in microfibril orientation within the secondary cell wall [43], and progressively develop different cell wall patterns during the maturation of protoxylem and then metaxylem vessel elements [44], the ability of the inner cortical cells within the Brassicaceae (but not *Arabidopsis thaliana*) to sequentially form two distinct secondary cell wall patterns within the same cell might be unique. Understanding the mechanisms that give rise to these different arrays might be instructive with regard to important questions about secondary wall development. Additionally, our measurements of JA’s effects on root growth and development, and its induction of phi thickenings and the reticulate network, are also important. By making detailed spatial measurements of where thickenings form, in comparison to other lignified structures such as the Casparian strip and protoxylem, we can demonstrate that 200 nM JA strongly induces thickenings while having only minor effects on root development, thus forming an important control for our initial biomechanical characterization of thickenings. And finally, the observed variability in phi thickening induction responses to JA [5] will allow for gene mapping experiments that will further dissect the pathways through which phi thickenings and the reticulate network develop.

## 4. Materials and Methods

### 4.1. Plant Material and Growth Conditions

*Brassica oleracea* L. cv. Marathon F1 (Mr Fothergill’s Seeds, South Windsor, NSW, Australia or Egmont Seeds, New Plymouth, New Zealand) were surface sterilized (50% (*v/v*) ethanol, 3% (*v/v*) hydrogen peroxide, 90 s), washed extensively with sterile water and plated onto quarter-strength MS media (1/4MS) in 1.2% agar (Becton-Dickinson, East Rutherford, NJ, USA) in 90 mm-diameter Petri dishes. After stratification for 24 to 48 h at 4 °C, seeds were germinated and grown for 3 to 5 days in a growth cabinet (16 h/8 h light/dark, 120 μmol·m^−2^s^−1^, 24 °C/20 °C) with the dishes oriented vertically. To induce phi thickenings, seedlings were grown on 1/4MS plates supplemented with jasmonic acid (JA) at either 200 nM, 1 μM or 10 μM.

### 4.2. Confocal Microscopy

Roots were fixed and imaged for lignin and cellulose [5]. Roots were fixed in phosphate-buffered saline (131 mM NaCl, 5.1 mM Na_2_HPO_4_, 1.56 mM KH_2_PO_4_, pH 7.2) supplemented with 3.7% formaldehyde, 1.0% (*v/v*) dimethyl sulfoxide and 0.1% (*v/v*) Triton X-100 (1 h or more). Washed roots were cleared in potassium hydroxide (10% (*w/v*), 60–80 °C overnight), washed in phosphate-buffered saline and then extracted in methanol (20 min). After lignin staining with basic fuchsin (0.01% (*w/v*), 2 min) and cell wall staining with calcofluor white (0.001% (*w/v*), 30 min) [5,10,45], samples were mounted in glycerol. Roots were imaged by confocal microscopy (Leica SP5, SP8) with calcofluor excited at 405 nm and collected from 420 to 460 nm, and with basic fuchsin excited concurrently at 561 nm with emissions collected from 580 to 700 nm. Z-stacks were collected with a step size of 1 to 1.5 µm, and the pinhole was minimized to optimize resolution in the Z-direction. Images were processed in ImageJ (version 1.52a) and Adobe Photoshop (version 27.11.0), with longitudinal section images shown as maximum projections covering ~50 µm in depth, while orthogonal (transverse) sections were generated using the reslice function in ImageJ and represent average projections covering ~5 µm. 3D movies were generated in ImageJ with the 3d project function.

### 4.3. Quantifying Root Elongation

After four days of growth, the location of the root tips was marked on the back of the plates, and after a further 24 h of growth, the plates were photographed. Changes in root length were measured in ImageJ using the segmented line function and used to calculate the root elongation rate.

### 4.4. Quantifying Root Development and Phi Thickenings

Because changes in cell length confounded our previous scoring system, based on cell counts [9,10], we now estimate phi thickening induction on a 0 to 5 scale, with 0 corresponding to no phi thickenings, and 5 corresponding to 60% or more coverage of thickenings [5]. Scoring was conducted with basic fuchsin-stained roots using widefield fluorescence imaging with a 10x lens and was conducted at the location in the root tip where phi thickening density was highest between the tip and the location at which the metaxylem had formed completely across the center of the root, a distance that was about 20 mm from the tip of control roots but closer to the tip in JA treatments.

To measure changes in root development on JA treatments, montages of fluorescence and transmitted light images of basic fuchsin-stained roots were prepared for the first 10 to 20 mm of the root. Root diameters were measured at four separate locations along the root and averaged, while the stele diameter was defined as the distance across the stele from one Casparian strip to the other, again measured at four different locations and averaged. Cell lengths were measured in inner cortical cells that contained phi thickenings: for control roots, these measurements were made in older regions where phi thickenings occasionally developed even under non-inducing conditions. The distance from the root tip to the first visible fluorescence from protoxylem and the lignified Casparian strip were measured, as were distances to the first visible phi thickenings and the formation of the first reticulate network. The location where phi thickenings first reached their highest density was also measured. Root measurements were made from nine or more replicate roots for the control and the three different JA treatments.

## Data Availability

All data presented in this study are available on request from the corresponding author.

## Supplementary Materials

The following supporting information can be downloaded at: https://www.mdpi.com/article/10.3390/plants15203162/s1; Figure S1: Woronin illustrated phi thickenings and the reticulate network in *Brassica* roots in 1878. Figure S2: Gaps in the phi thickening network can remain following root development and differentiation. Video S1 The development of multiple rings of phi thickenings. Video S2: The development of multiple rings of phi thickenings. Video S3: Casparian strip and phi thickening organization during lateral root development.

## Abbreviations

The following abbreviations are used in this manuscript:

1/4MS	quarter-strength MS media
3D	three dimensional
ABA	abscisic acid
JA	jasmonic acid
LRR-RK	leucine-rich repeat receptor kinase

## References

[1] Van Tieghem P. (1888). Sur le réseau de soutien de l’ecorce de la racine. Ann. Sci. Nat. Ser. 7.

[2] Fernández-García N., López-Berenguer C., Olmos E., Morte A., Varma A. (2014). Role of phi cells under abiotic stress in plants. Root Engineering.

[3] Aleamotuʻa M., McCurdy D.W., Collings D.A. (2019). Phi thickenings in roots: Novel secondary wall structures responsive to biotic and abiotic stresses. J. Exp. Bot..

[4] Collings D.A., Aleamotuʻa M., McCurdy D.W., Cánovas F.M., Lüttge U., Leuschner C., Risueño M.-C. (2021). Phi thickenings: Their history, current status and presumed role(s) in mechanically strengthening the plant root. Progress in Botany.

[5] Collings D.A., Aleamotuʻa M., McCurdy D.W. (2026). Structural conservation of phi thickenings in diverse plant taxa. Ann. Bot..

[6] Nicolai O. (1865). Das Wachsthum der Wurzel. Schriften. Königl. Phys.-Ökon. Ges. Königsberg..

[7] Van Tieghem P. (1871). Mémoire sur la racine. Ann. Sci. Nat. Ser. 5.

[8] Woronin M. (1878). *Plasmodiophora brassicae*, Urheber der Kohl-pflanzenhernie. Jahrb. Wiss. Bot..

[9] Aleamotuʻa M., Baker J.K., McCurdy D.W., Collings D.A. (2022). Phi thickenings in *Brassica oleracea* roots are induced by osmotic stress and mechanical effects, both involving jasmonic acid. J. Exp. Bot..

[10] Aleamotuʻa M., Tai Y.-T., McCurdy D.W., Collings D.A. (2018). Developmental biology and induction of phi thickenings by abiotic stress in roots of the Brassicaceae. Plants.

[11] Van Tieghem P. (1887). Sur le réseau sous-endodermique de la racine des cruciferes. Bull. Soc. Bot. Fr..

[12] van de Mortel J.E., Almar Villanueva L., Schat H., Kwekkeboom J., Coughlan S., Moerland P.D., Ver Loren van Themaat E., Koornneef M., Aarts M.G.M. (2006). Large expression differences in genes for iron and zinc homeostasis, stress response, and lignin biosynthesis distinguish roots of *Arabidopsis thaliana* and the related metal hyperaccumulator *Thlaspi caerulescens*. Plant Physiol..

[13] Zelko I., Lux A., Czibula K. (2008). Difference in the root structure of hyperaccumulator *Thlaspi caerulescens* and non-hyperaccumulator *Thlaspi arvense*. Int. J. Environ. Pollut..

[14] Kováč J., Lux A., Soukup M., Weidinger M., Gruber D., Lichtscheidl I., Vaculík M. (2020). A new insight on structural and some functional aspects of peri-endodermal thickenings, a specific layer in *Noccaea caerulescens* roots. Ann. Bot..

[15] Scialabba A., Melati M.R. (1990). The effect of NaCl on growth and xylem differentiation of radish seedlings. Bot. Gaz..

[16] López-Pérez L., Fernández-García N., Olmos E., Carvajal M. (2007). The phi thickening in roots of broccoli plants: An acclimation mechanism to salinity?. Int. J. Plant Sci..

[17] Fernández-García N., Lopez-Perez L., Hernandez M., Olmos E. (2009). Role of phi cells and the endodermis under salt stress in *Brassica oleracea*. New Phytol..

[18] Weerdenburg C., Peterson C.A. (1983). Structural changes in phi thickenings during primary and secondary growth in roots. 1. Apple (*Pyrus malus*) Rosaceae. Can. J. Bot..

[19] Vilches-Barro A., Maizel A. (2015). Talking through walls: Mechanisms of lateral root emergence in *Arabidopsis thaliana*. Curr. Opin. Plant Biol..

[20] Yalamanchili K., Vermeer J.E.M., Scheres B., Willemsen V. (2024). Shaping root architecture: Towards understanding the mechanisms involved in lateral root development. Biol. Direct.

[21] Cui H. (2016). Middle cortex formation in the root: An emerging picture of integrated regulatory mechanisms. Mol. Plant.

[22] Liu J., Yu Z., Ding Z. (2024). Root development: A new player integrates two old friends. Curr. Biol..

[23] Bonacorsi N.K., Seago J.L. (2016). Root development and structure in seedlings of *Ginkgo biloba*. Am. J. Bot..

[24] Scott L.I., Whitworth A.B. (1928). On a structural peculiarity of the exodermis of the root of *Pelargonium*. Proc. Leeds Philos. Lit. Soc..

[25] Haas D.L., Carothers Z.B., Robbins R.R. (1976). Observations on the phi- thickenings and Casparian strips in *Pelargonium* roots. Am. J. Bot..

[26] Peterson C.A., Emanuel M.E., Weerdenburg C.A. (1981). The permeability of phi thickenings in apple (*Pyrus malus*) and geranium (*Pelargonium hortorum*) roots to an apoplastic fluorescent dye tracer. Can. J. Bot..

[27] Vermeer J.E., von Wangenheim D., Barberon M., Lee Y., Stelzer E.H., Maizel A., Geldner N. (2014). A spatial accommodation by neighboring cells is required for organ initiation in *Arabidopsis*. Science.

[28] Peterson C.A., Lefcourt B.E.M. (1990). Development of endodermal Casparian bands and xylem in lateral roots of broad bean. Can. J. Bot..

[29] von Wangenheim D., Goh T., Dietrich D., Bennett M.J. (2017). Plant biology: Building barriers… in roots. Curr. Biol..

[30] Serra O., Geldner N. (2022). The making of suberin. New. Phytol..

[31] Sharma A., Sinharoy S., Bisht N.C. (2023). The mysterious non-arbuscular mycorrhizal status of *Brassicaceae* species. Environ. Microbiol..

[32] Schwendener S. (1874). Das Mechanische Princip in Anatomischen Bau der Monocotylen: Mit Vergleichenden Ausblicken auf Übrigen Pflanzenklassen.

[33] Doblas V.G., Smakowska-Luzan E., Fujita S., Alassimone J., Barberon M., Madalinski M., Belkhadir Y., Geldner N. (2017). Root diffusion barrier control by a vasculature-derived peptide binding to the SGN3 receptor. Science.

[34] Nakayama T., Shinohara H., Tanaka M., Baba K., Ogawa-Ohnishi M., Matsubayashi Y. (2017). A peptide hormone required for Casparian strip diffusion barrier formation in *Arabidopsis* roots. Science.

[35] Shinohara H., Mori A., Yasue N., Sumida K., Matsubayashi Y. (2016). Identification of three LRR-RKs involved in perception of root meristem growth factor in *Arabidopsis*. Proc. Natl. Acad. Sci. USA.

[36] Laffont C., Ivanovici A., Gautrat P., Brault M., Djordjevic M.A., Frugier F. (2020). The NIN transcription factor coordinates CEP and CLE signaling peptides that regulate nodulation antagonistically. Nat. Commun..

[37] Ito M., Tajima Y., Ogawa-Ohnishi M., Nishida H., Nosaki S., Noda M., Sotta N., Kawade K., Kamiya T., Fujiwara T. (2024). IMA peptides regulate root nodulation and nitrogen homeostasis by providing iron according to internal nitrogen status. Nat. Commun..

[38] Okada K., Kubota Y., Hirase T., Otani K., Goh T., Hiruma K., Saijo Y. (2021). Uncoupling root hair formation and defence activation from growth inhibition in response to damage-associated Pep peptides in *Arabidopsis thaliana*. New Phytol..

[39] Schoenaers S., Lee H.K., Gonneau M., Faucher E., Levasseur T., Akary E., Claeijs N., Moussu S., Broyart C., Balcerowicz D. (2024). Rapid alkalinization factor 22 has a structural and signalling role in root hair cell wall assembly. Nat. Plants.

[40] Smakowska-Luzan E., Mott G.A., Parys K., Stegmann M., Howton T.C., Layeghifard M., Neuhold J., Lehner A., Kong J., Grünwald K. (2018). An extracellular network of *Arabidopsis* leucine-rich repeat receptor kinases. Nature.

[41] Oh E.-S., Lee Y., Chae W.B., Rameneni J.J., Park Y.-S., Lim Y.P., Oh M.-H. (2018). Biochemical analysis of the role of leucine-rich repeat receptor-like kinases and the carboxy-terminus of receptor kinases in regulating kinase activity in *Arabidopsis thaliana* and *Brassica oleracea*. Molecules.

[42] Song M., Linghu B., Huang S., Li F., An R., Xie C., Zhu Y., Hu S., Mu J., Zhang Y. (2022). Genome-wide survey of leucine-rich repeat receptor-like protein kinase genes and CRISPR/Cas9-targeted mutagenesis *BnBRI1* in *Brassica napus*. Front. Plant Sci..

[43] Donaldson L.A. (2019). Wood cell wall ultrastructure. The key to understanding wood properties and behaviour. IAWA J..

[44] Xu H., Giannetti A., Sugiyama Y., Zheng W., Schneider R., Watanabe Y., Oda Y., Persson S. (2022). Secondary cell wall patterning—Connecting the dots, pits and helices. Open Biol..

[45] Kapp N., Barnes W.J., Richard T.L., Anderson C.T. (2015). Imaging with the fluorogenic dye basic fuchsin reveals subcellular patterning and ecotype variation of lignification in *Brachypodium distachyon*. J. Exp. Bot..

