# Supplementary material for: The Anatomy of Phi Thickening Development in Brassica oleracea Roots
Source: Plants (Basel). 2026 Oct 15;15(20):3162. doi: 10.3390/plants15203162 (PMC13708876; doi:10.3390/plants15203162)
Supplement: Supplementary file 1 [file plants-15-03162-s001.zip › plants-4596533-supplementary/COLLINGS - supplementary data/COLLINGS - supplementary figures.pdf]

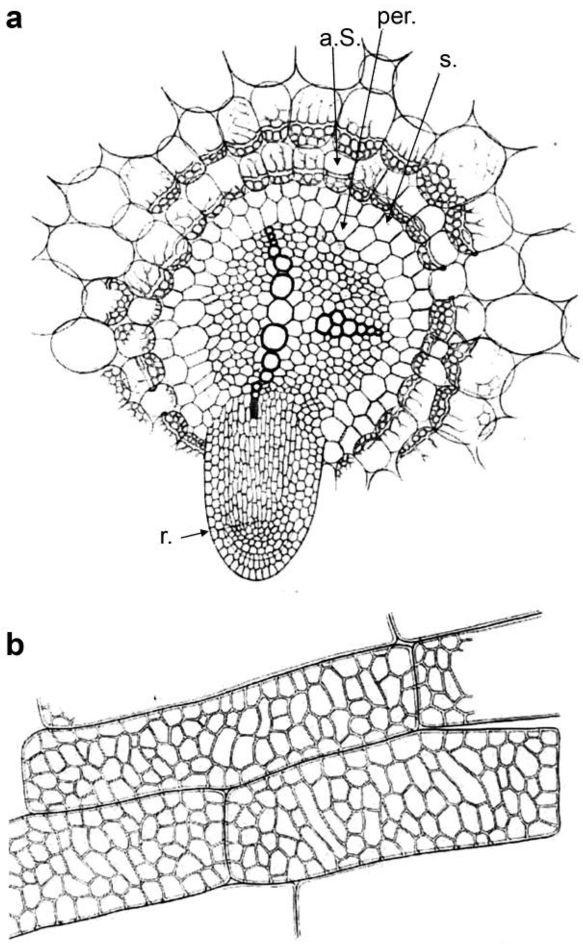

Figure S1. Woronin illustrated phi thickenings and the reticulate network in *Brassica* roots in 1878. The first descriptions and illustrations of both phi thickenings in *Brassica*, including in multiple layers, and the reticulate network were made by Woronin in 1878, and published in ***J****ahrbücher für Wissenschaftliche Botanik* [8]. This paper is available in the public domain through a scan of the original at the University of California (https://babel.hathitrust.org/cgi/pt?id=uc1.a0013801253&seq=735). These images are reprinted here with the text of the original German figure legends, translated into English, shown in italics. Labeling on the figures follows the original images has been refreshed. Note that while the multiple layers of phi thickenings and reticulate network are indicated, the Casparian strip itself is not shown within the endodermal cells. Also note that the root is shown as triarch, with three xylem poles, whereas *Brassica* roots are typically diarch with two. xylem poles opposite one another.

a *Central section of a cross-section through a somewhat older root. The characteristic thickenings that occur in the cells of the innermost periphery layer (a.S.) are particularly visible in this section. per = pericycle (the rhizogenous layer following van Tieghem), s = the endodermis (protective layer following Caspary), r = young lateral root growing out of the pericycle.* **b** *Cells of the innermost periphery layer on the tangential longitudinal section, observed from the inner side, with which they are directly attached to the endodermis.*

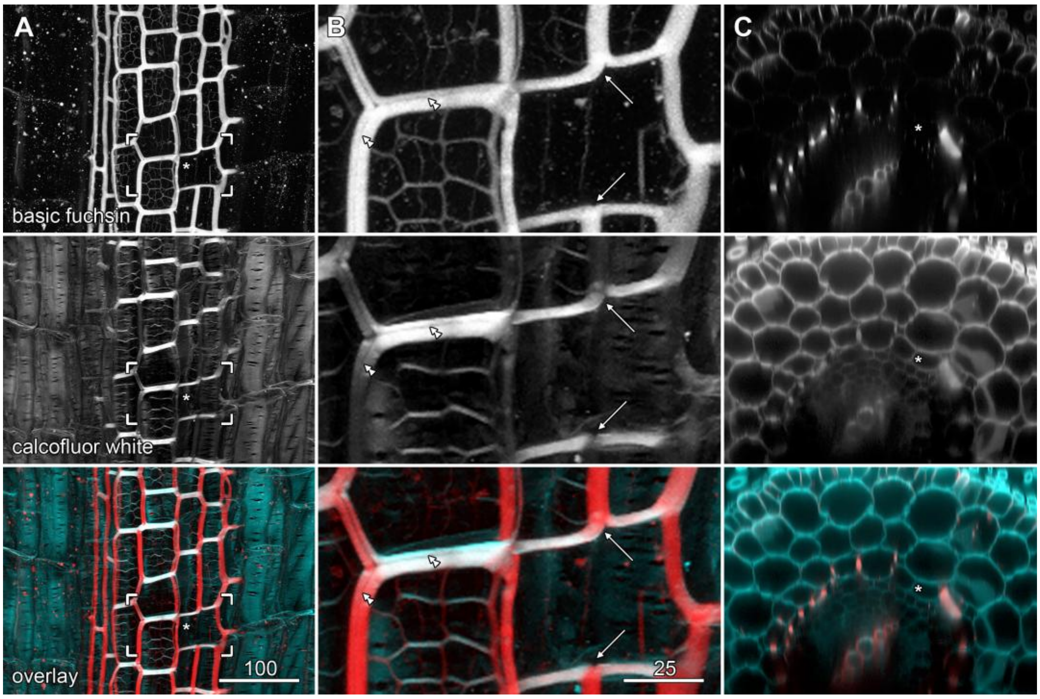

Figure S2. Gaps in the phi thickening network can remain following root development and differentiation. Roots grown on 1 μM JA for 5 days were stained with basic fuchsin for lignin (red in overlay) and calcofluor white for cell walls (cyan in overlay) and imaged ~25 mm from the root tip by confocal microscopy. Images are shown as longitudinal sections (**A**, **B**), with the area boxed in **A** shown at higher magnification in panel **B**, or orthogonal (cross) images calculated in ImageJ (**C**). Two adjacent cells that lack phi thickenings are indicated (asterisks) in which cell wall bands occur only in the adjacent cells leading to indentations in the bands at the sites of cell junctions (arrows), and an absence of a visible middle lamella, present in adjacent cells (double headed arrowheads). Bar in **A** = 100 μm for **A** and **C**; bar in **B** = 25 μm.

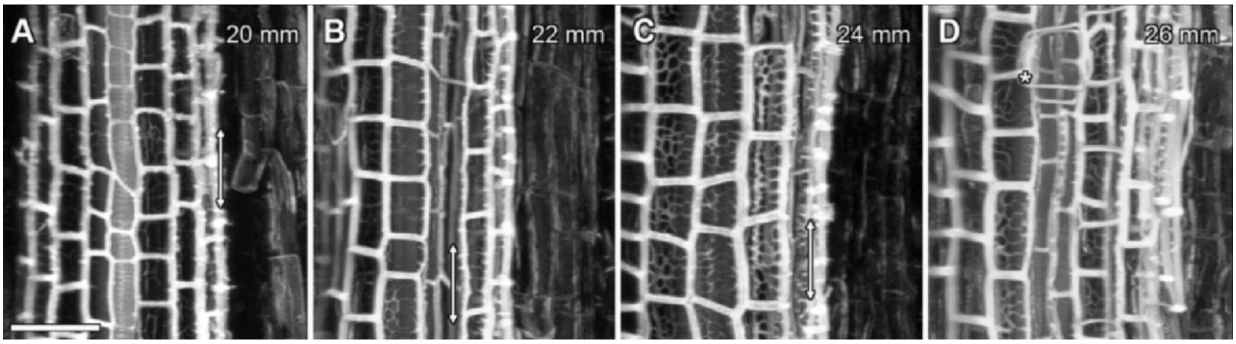

Video S1. The development of multiple rings of phi thickenings. The double-headed arrows indicate extra files of thickenings internal to the complete phi thickening ring, while the asterisks mark single cells with thickenings that are external to the general phi thickening layer. Scale bar = 100 μm for all images. See Figure 3.

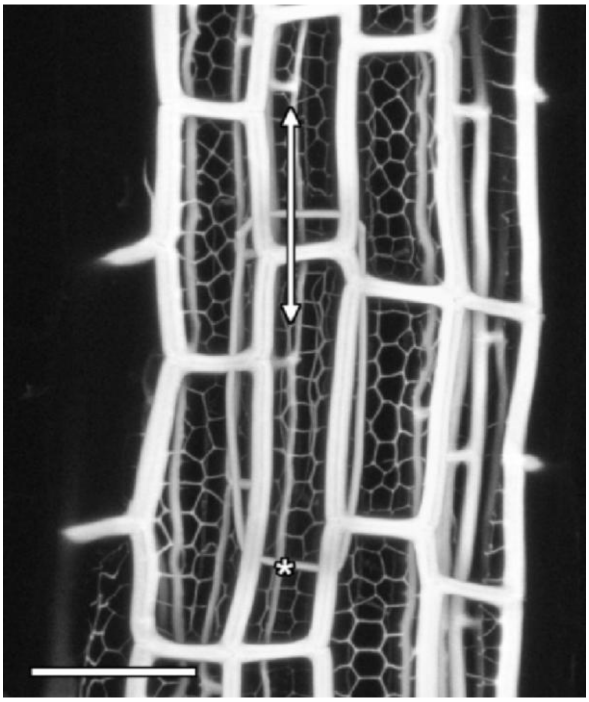

Video S2. The development of multiple rings of phi thickenings. The asterisk marks a single extra cell with phi thickenings while the double-headed arrow shows extra files of phi thickening-containing cells internal to the main ring of phi thickenings. Scale bar = 100 μm. See Figure 4.

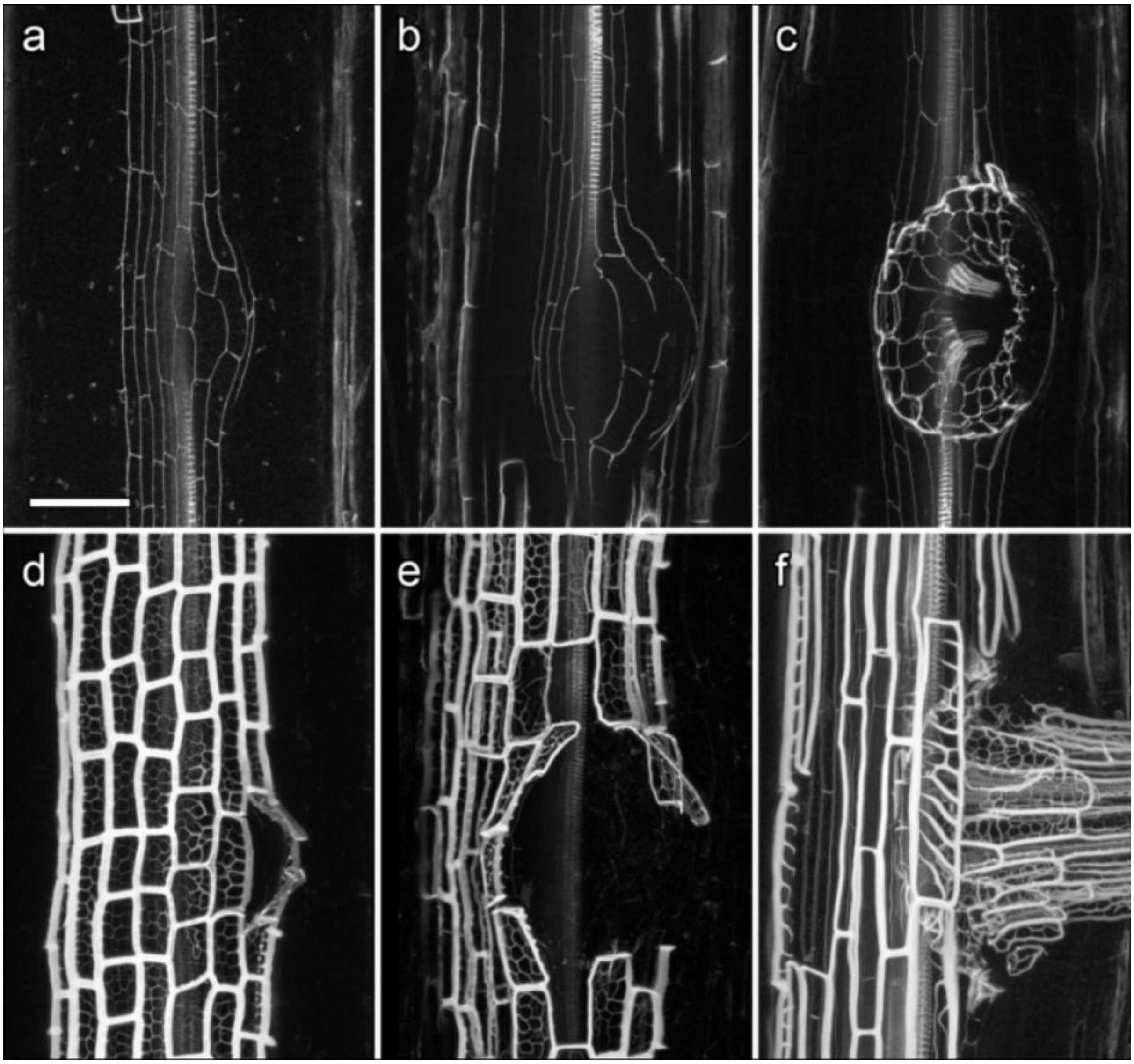

Video S3. Casparian strip and phi thickening organization during lateral root development. **a**-**c** Uninduced root. **d**-**f** Root grown on JA (1 μM). **a**, **d** Lateral root initial only just formed. Fractures in the lignified Casparian strip had already developed (asterisks). **b**, **e** Lateral root extension to the surface of the primary root. **c**, **f** Older lateral root. Note that the phi thickenings in the lateral root form a separate network from the phi thickenings in the primary root, separated by a gap in which the only lignified tissue is the Casparian strip ring. Scale bar in **a** = 100 μm for all images. See Figure 5.
